# Supplementary material for: NMDAR‐dependent Argonaute 2 phosphorylation regulates miRNA activity and dendritic spine plasticity
Source: EMBO J. 2018 Apr 30;37(11):e97943. doi: 10.15252/embj.201797943 (PMC5983126; doi:10.15252/embj.201797943)
Supplement: Supplementary file 1 — Appendix [file EMBJ-37-e97943-s001.pdf]

Appendix.

Table of Contents.

P.2 - Appendix Figure S1. Characterization of Ago2 molecular replacement constructs.
Related to Figure 3.

P.3 - Appendix Figure S2. Dissociation of PICK1 from Ago2 is not required for NMDA-induced S387 phosphorylation.
Related to Figure 3.

P.4 - Appendix Figure S3. Ago2 knockdown does not cause a change in Ago1 or Ago3 expression, and Ago1 does
not recue functional deficits caused by Ago2 knockdown.
Related to Figure 5.

P. 5- Appendix Figure S4. Comparison between Luciferase reporters incorporating WT 3'UTRs and those carrying mutations to block miRNA binding.
Related to Figure 5.

Appendix Figure S1. Characterization of Ago2 molecular replacement constructs. Related to Figure 3.

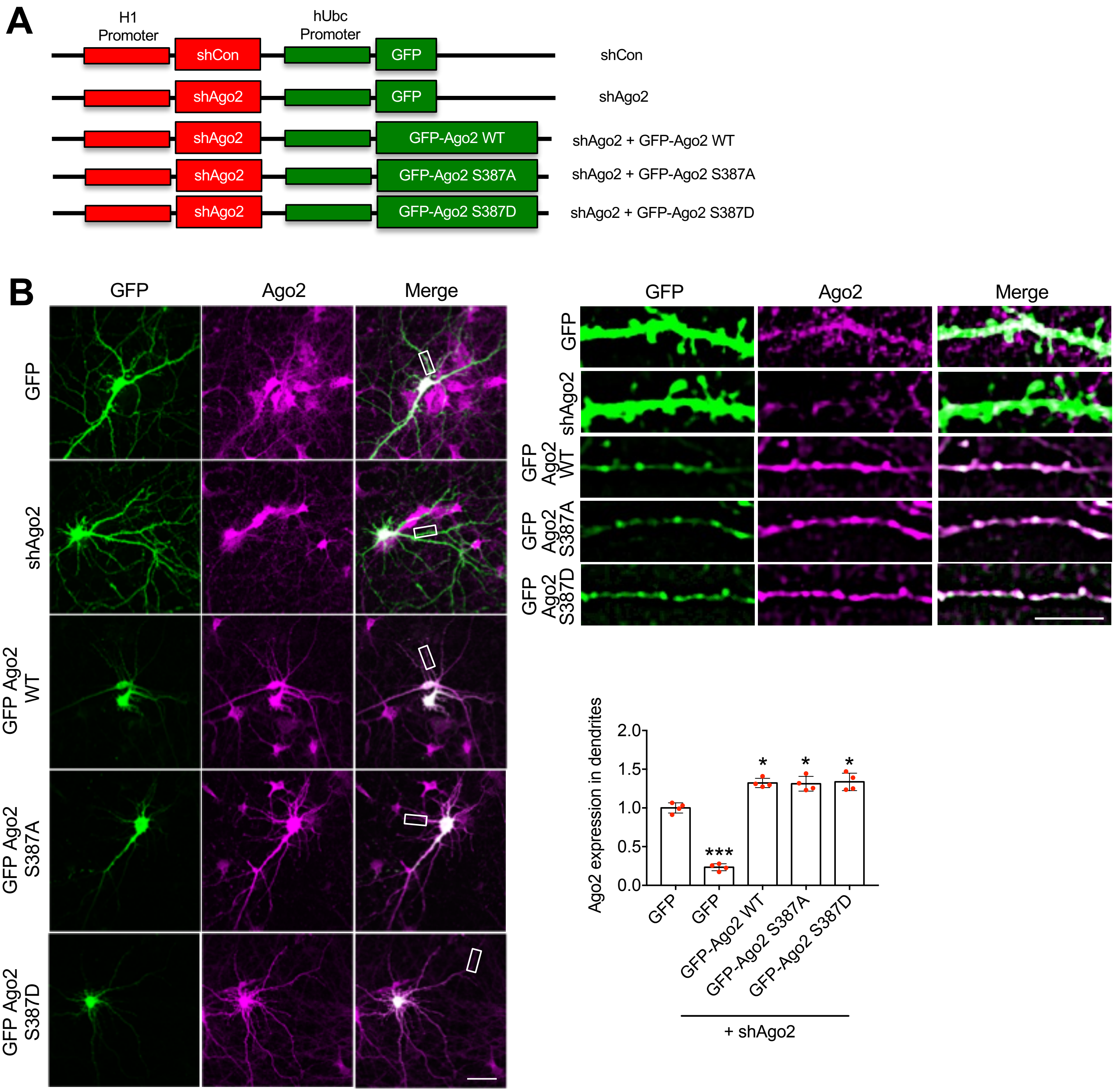

A) Schematic illustration of Ago2 molecular replacement constructs cloned into FUGW vector. H1 promoter drives expression of control or Ago2 shRNA and the ubiquitin (Ubi) promoter drives expression of GFP, GFP-Ago2 WT, GFP-Ago2 S387A or GFP-Ago2 S387D.

B) Cultured cortical neurons were transfected with the molecular replacement constructs depicted in A. Five days after transfection, cells were fixed, permeabilized and stained with Ago2 antibodies. Images are representative dendrites showing GFP fluorescence (green) and Ago2 staining (magenta). Graph shows quantification of Ago2 staining intensity in neuronal dendrites. Scale bar = 50µ for whole cell images and 20µ for dendrite images; n= 8 cells from four independent experiments, *$p<0.05$, ***$p<0.001$, one-way ANOVA, Bonferroni post hoc test. Ago2 shRNA knocks down endogenous Ago2 by ~80%, whilst constructs expressing Ago2 shRNA + GFP-Ago2 result in a slight over-rescue of Ago2 expression by ~30%.

Appendix Figure S2. Dissociation of PICK1 from Ago2 is not required for NMDA-induced S387 phosphorylation.
Related to Figure 3.

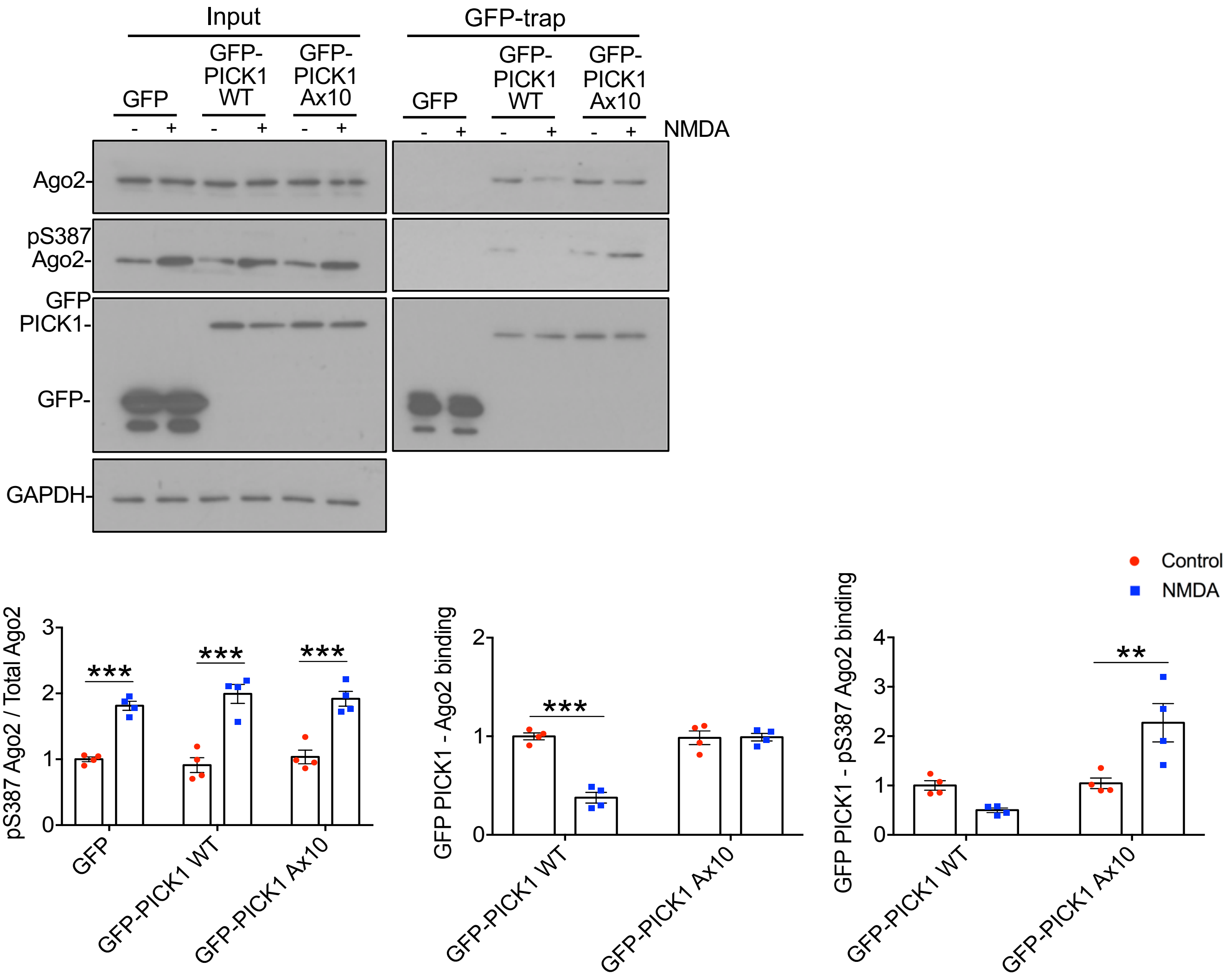

Cultured neurons were transfected with GFP or PICK1 molecular replacement constructs expressing PICK1 shRNA plus sh-resistant GFP-PICK1-WT or GFP-PICK1-Ax10. Lysates were prepared 6 min after NMDA washout, and GFP-PICK1 complexes were precipitated using GFP-trap beads. Bound proteins were detected by Western blotting using Ago2, pS387-Ago2 or GFP antibodies as shown. Graphs show quantification of pS387 Ago2/ total Ago2 ratio normalized to untreated GFP condition (left), GFP-PICK1 binding to Ago2, normalized to untreated PICK1-WT condition (middle), and GFP-PICK1 binding to pS387 Ago2, normalized to untreated PICK1-WT condition (right); n=4, **p<0.01; ***p<0.001, two-way ANOVA, Bonferroni post hoc test.

Appendix Figure S3. Ago2 knockdown does not cause a change in Ago1 or Ago3 expression, and Ago1 doe not recue functional deficits caused by Ago2 knockdown.
Related to Figure 5.

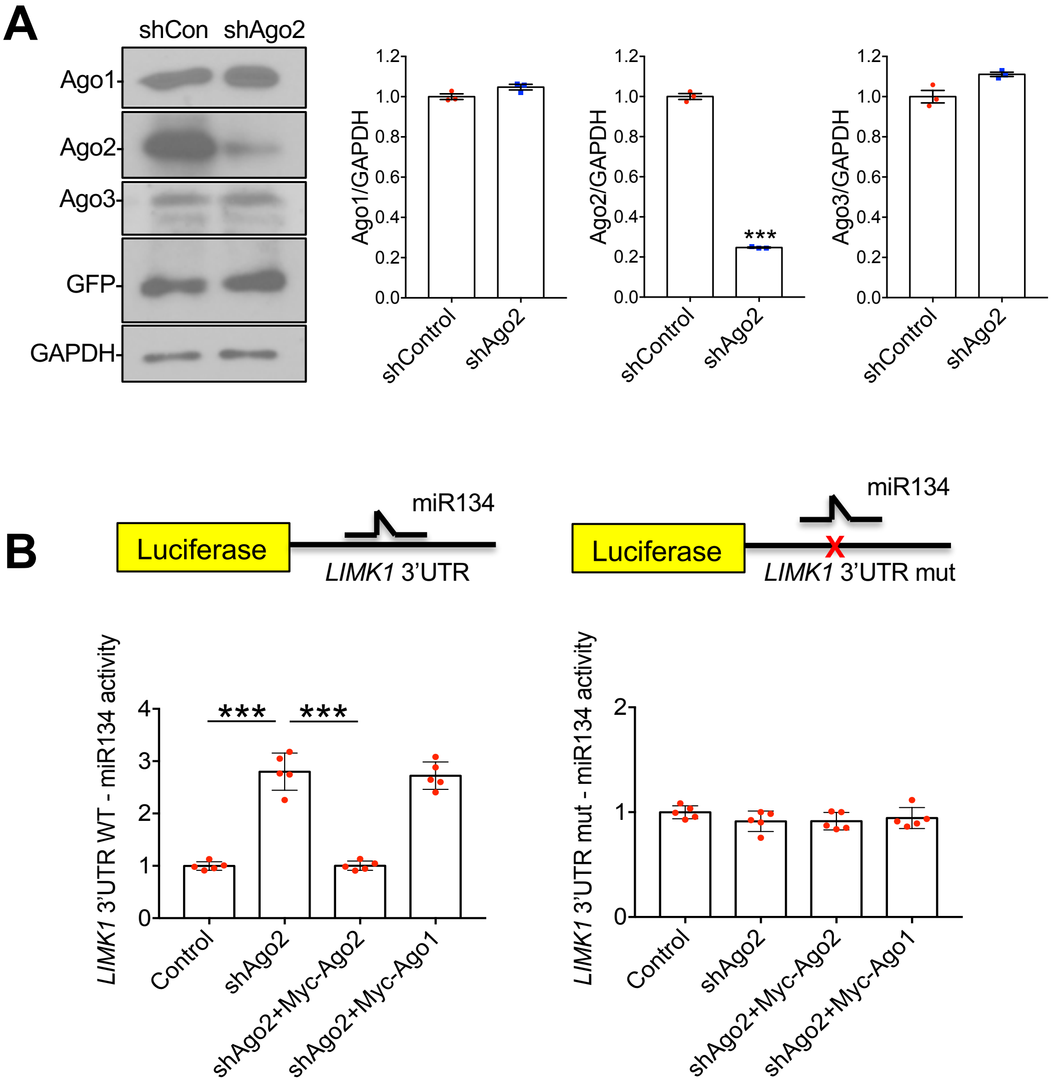

A) Ago2 knockdown does not cause changes in Ago1 or Ago3 expression level. Cultured neurons were transduced with lentivirus expressing Ago2 shRNA plus GFP. 7 days after transduction, lysates were analysed by Western blotting using antibodies as shown. Graphs show quantification of Ago1, Ago2 and Ago3 levels, normalised to GAPDH. N=3, ***p<0.001, t-test.

B) The loss of translational repression of *LIMK1* via miR-134 caused by Ago2 knockdown is rescued by Ago2, but not Ago1. Cultured neurons were transfected with Ago2 shRNA, sh-resistant ^{myc}Ago2, or ^{myc}Ago1, as well as Renilla luciferase and Firefly luciferase reporters containing *LIMK1* 3'UTR (left) or *LIMK1* 3'UTR containing mutations in the seed region for miR-134 (right). ***p<0.001, one-way ANOVA, Bonferroni post hoc test.

Appendix Figure S4. Comparison between Luciferase reporters incorporating WT 3'UTRs and those carrying mutations to block miRNA binding.
Related to Figure 5.

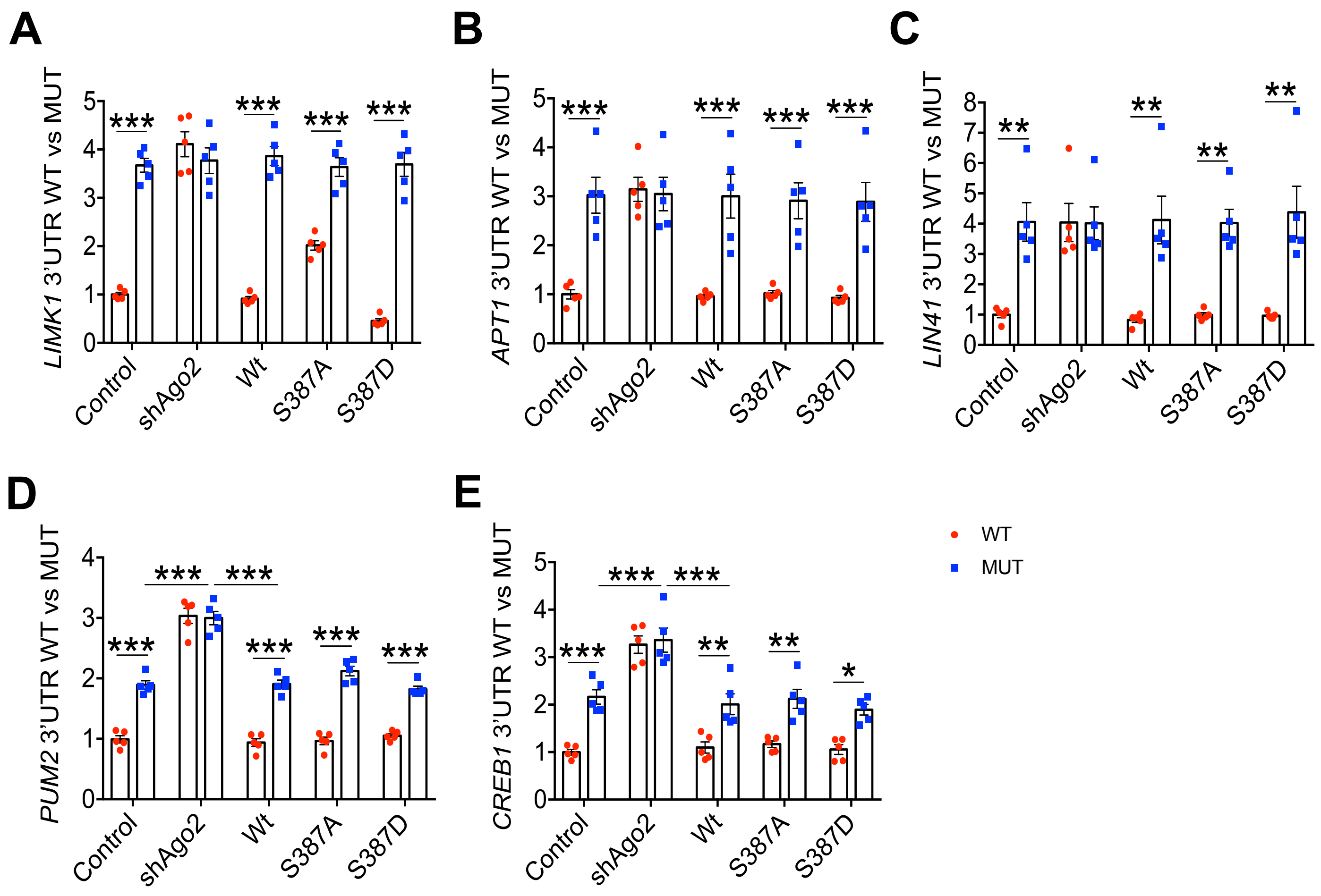

Luciferase data from Figure 5 and Supplementary Figure S6 analysed as comparisons between constructs incorporating WT 3'UTRs and miR-134 seed region mutant 3'UTRs. n=5, *p<0.05; **p<0.01; ***p<0.001, two-way ANOVA, Bonferroni post hoc test.
