# Supplementary material for: NMDAR‐dependent Argonaute 2 phosphorylation regulates miRNA activity and dendritic spine plasticity
Source: EMBO J. 2018 Apr 30;37(11):e97943. doi: 10.15252/embj.201797943 (PMC5983126; doi:10.15252/embj.201797943)
Supplement: Supplementary file 2 — Expanded View Figures PDF [file EMBJ-37-e97943-s002.pdf]

# Expanded View Figures

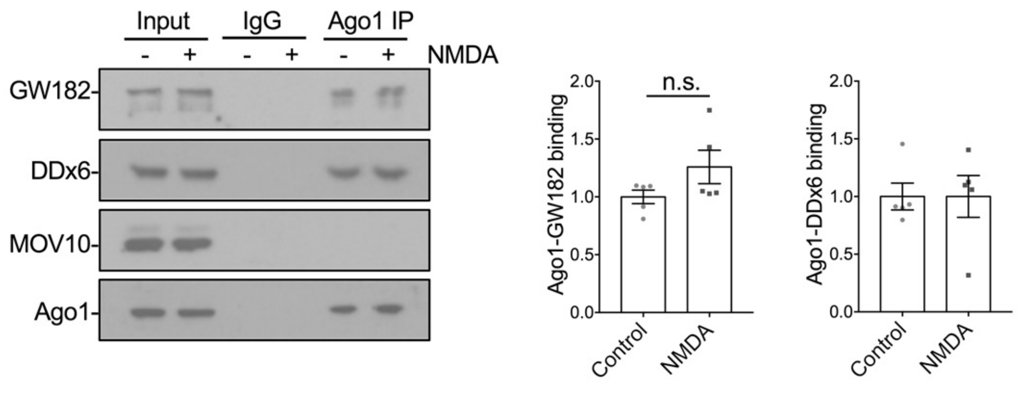


**Figure EV1. Endogenous Ago1-GW182 and Ago1-DDX6 interactions are unaffected by NMDAR stimulation (related to Fig 1).**

Cortical neuronal cultures were exposed to NMDA or vehicle for 3 min; lysates were prepared 10 min after NMDA washout and immunoprecipitated with Ago1 antibodies. Proteins were detected by Western blotting. Graph shows quantification of Ago1-GW182 interaction, normalised to vehicle control; $n = 5$; $t$-test.

Source data are available online for this figure.

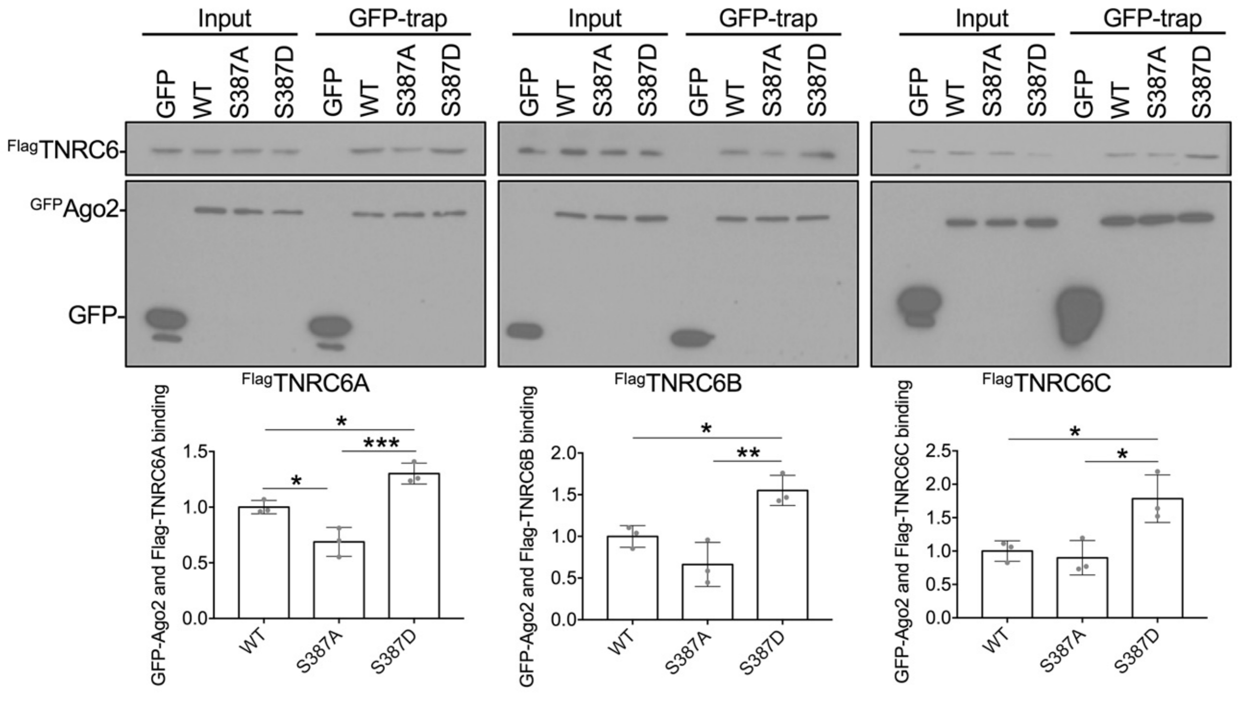


**Figure EV2. Ago2 binding to all three GW proteins TNRC6A-C is affected by Ago2 S387 mutations (related to Fig 3).**

HEK293 cells transfected with Flag-tagged TNRC6A, B or C plus GFP, GFP-WT-Ago2, GFP-S387A-Ago2 or GFP-S387D-Ago2 were lysed, and GFP-Ago2 complexes were precipitated using GFP-trap beads. Bound proteins were detected by Western blotting using Flag or GFP antibodies as shown. Graphs show quantification of GFP-Ago2 interactions with TNRC6 isoforms, normalised to GFP-WT-Ago2; $n = 3$. *$P < 0.05$, **$P < 0.01$, ***$P < 0.001$; two-way ANOVA, Bonferroni *post hoc* test. Error bars are SEM.

Source data are available online for this figure.

 © 2018 The Authors

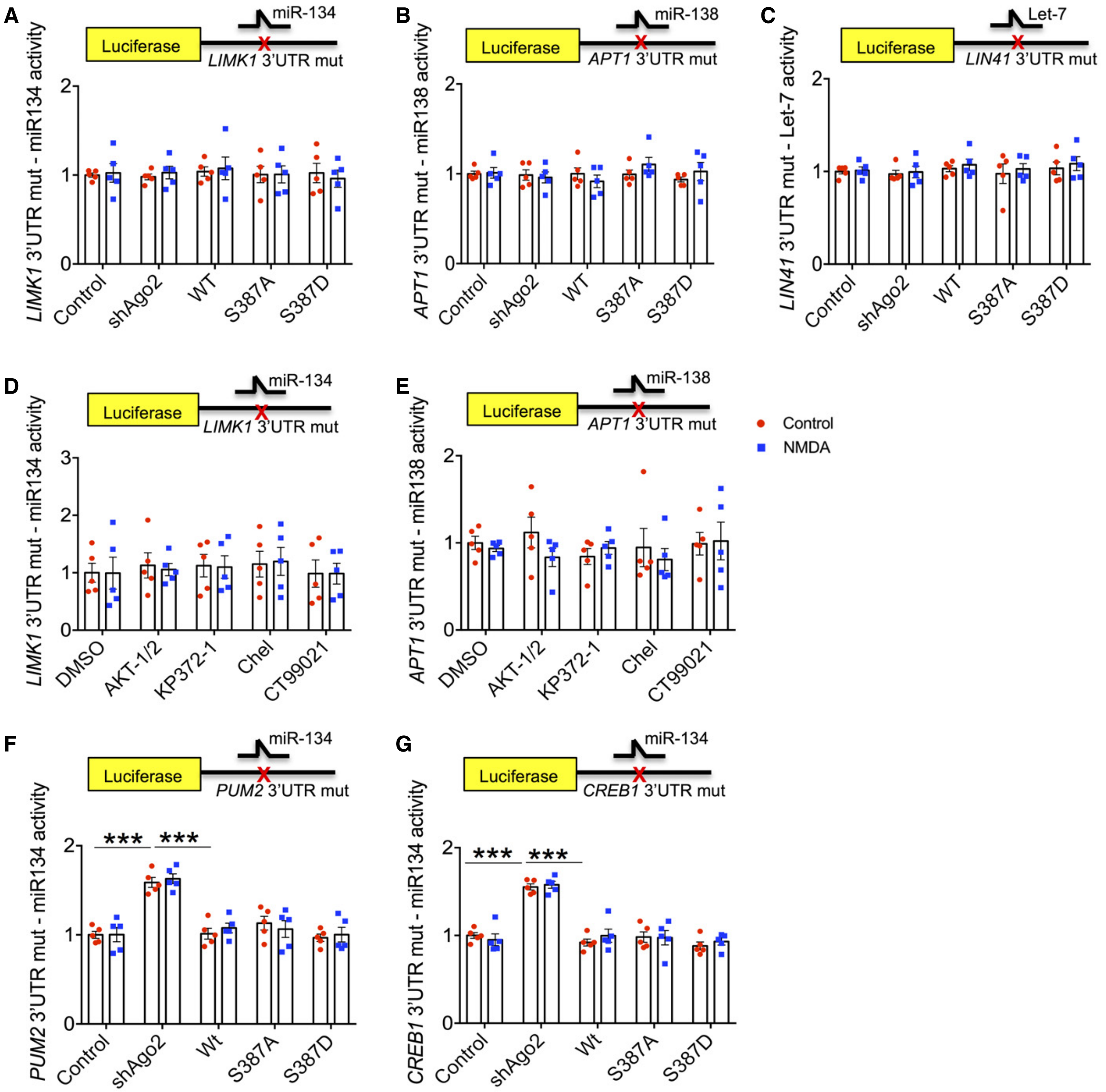


**Figure EV3. Luciferase assays using 3′UTRs carrying mutations to block miRNA binding (related to Fig 5).**

A–E The effects on luciferase reporters incorporating *LIMK1*, *APT1* and *LIN41* 3′UTRs seen in Fig 5 are fully explained by regulation via miR-134, miR-138 and Let-7, respectively. Cultured cortical neurons transfected with Ago2 molecular replacement constructs as well as *Renilla* luciferase and *Firefly* luciferase reporters containing LIMK1 (A, D), APT1 (B, E) or LIN41 (C) 3′UTRs containing mutations in the seed regions for miR-134, miR-138 and Let-7, respectively, were treated with NMDA or vehicle for 3 min. Ten minutes after NMDA washout, lysates were prepared for dual-luciferase assays. For (D, E), cultures were also treated with kinase inhibitors as shown 20 min before NMDA or vehicle. Error bars are SEM.

F, G Translation of luciferase reporters incorporating *PUM2* or *CREB1* is regulated by other miRNAs in addition to miR-134. Experiment was performed as in (A), except *Firefly* luciferase reporters contained *PUM2* (F), *CREB1* (G) 3′UTRs carrying mutations in the seed regions for miR-134. $n = 5$. ***$P < 0.001$, two-way ANOVA, Bonferroni *post hoc* test. Error bars are SEM.

© 2018 The Authors

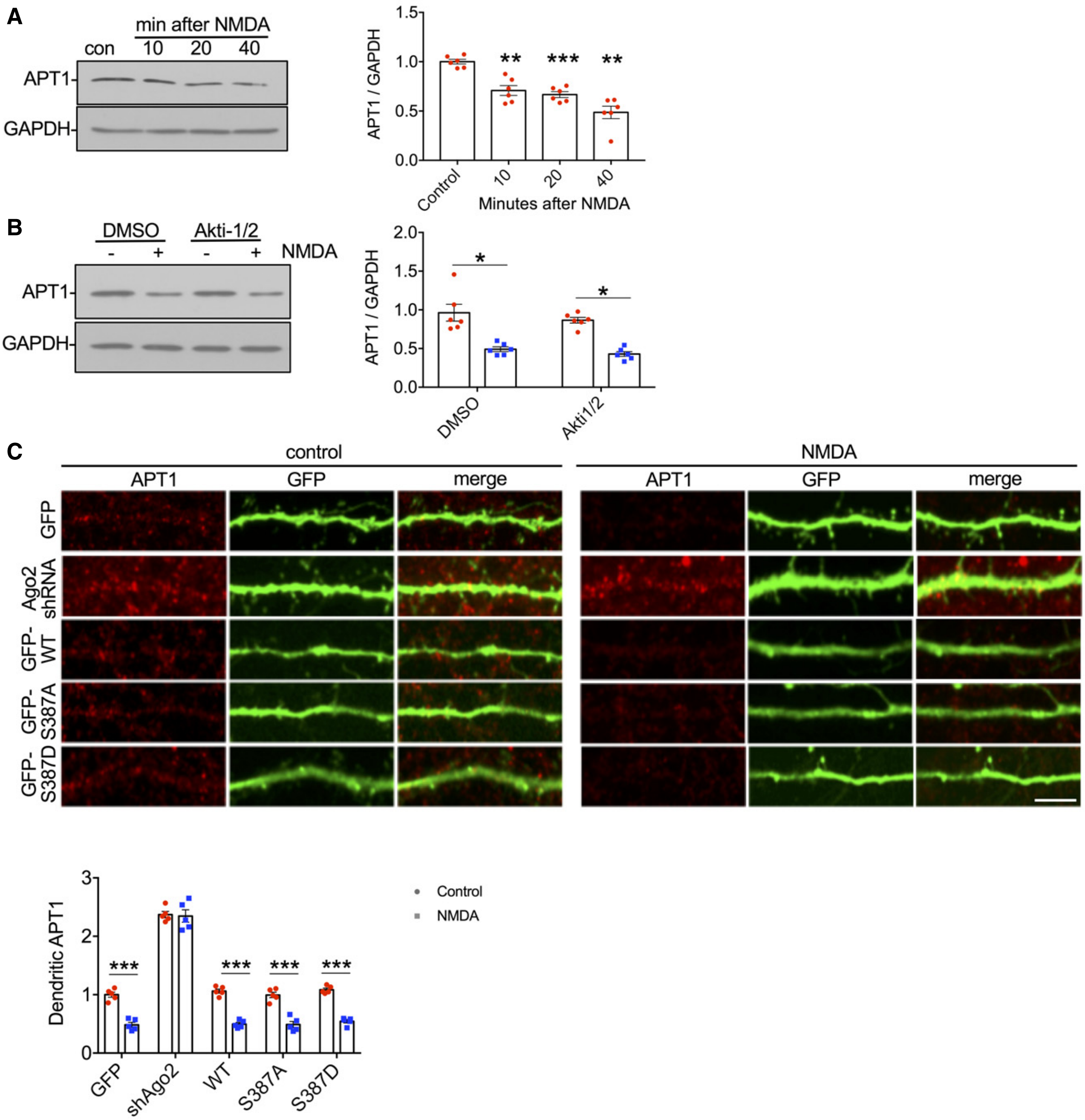


**Figure EV4. NMDAR-dependent decrease in APT1 expression is insensitive to Akt inhibition or Ago2 phosphorylation at S387 (related to Fig 7).**

A Endogenous APT1 protein levels are rapidly reduced in response to NMDAR stimulation. Cortical neuronal cultures were exposed to NMDA or vehicle for 3 min, and lysates were prepared 10, 20 or 40 min after NMDA washout and analysed by Western blotting. Graphs show quantification of APT1 expression normalised to vehicle control; $n = 6$. $^{**}P < 0.01$; $^{***}P < 0.001$ one-way ANOVA, Bonferroni *post hoc* test. Error bars are SEM.

B NMDAR-dependent decrease in APT1 is Akt-independent. Cortical neuronal cultures were treated with Akti-1/2 20 min before NMDA or vehicle application, and lysates were prepared 40 min after NMDA washout and analysed by Western blotting. Graphs show quantification of APT1 expression normalised to vehicle control; $n = 6$. $^{*}P < 0.05$; two-way ANOVA, Bonferroni *post hoc* test. Error bars are SEM.

C NMDAR-dependent decrease in dendritic APT1 expression is unaffected by Ago2 phosphorylation at S387. Cortical neurons were transfected with molecular replacement constructs expressing Ago2 shRNA plus shRNA-resistant GFP-Ago2 (WT, S387A or S387D), fixed 40 min after NMDA washout, permeabilised and stained with APT1 antibodies (red channel). GFP signal was maximised at acquisition so that dendrites could be effectively visualised. Graph shows APT1 staining intensity in dendrites normalised to vehicle control. Scale bar = 20 μm; $n = 10$ cells from five independent experiments. $^{***}P < 0.001$ two-way ANOVA, Bonferroni *post hoc* test. Error bars are SEM.

Source data are available online for this figure.


© 2018 The Authors

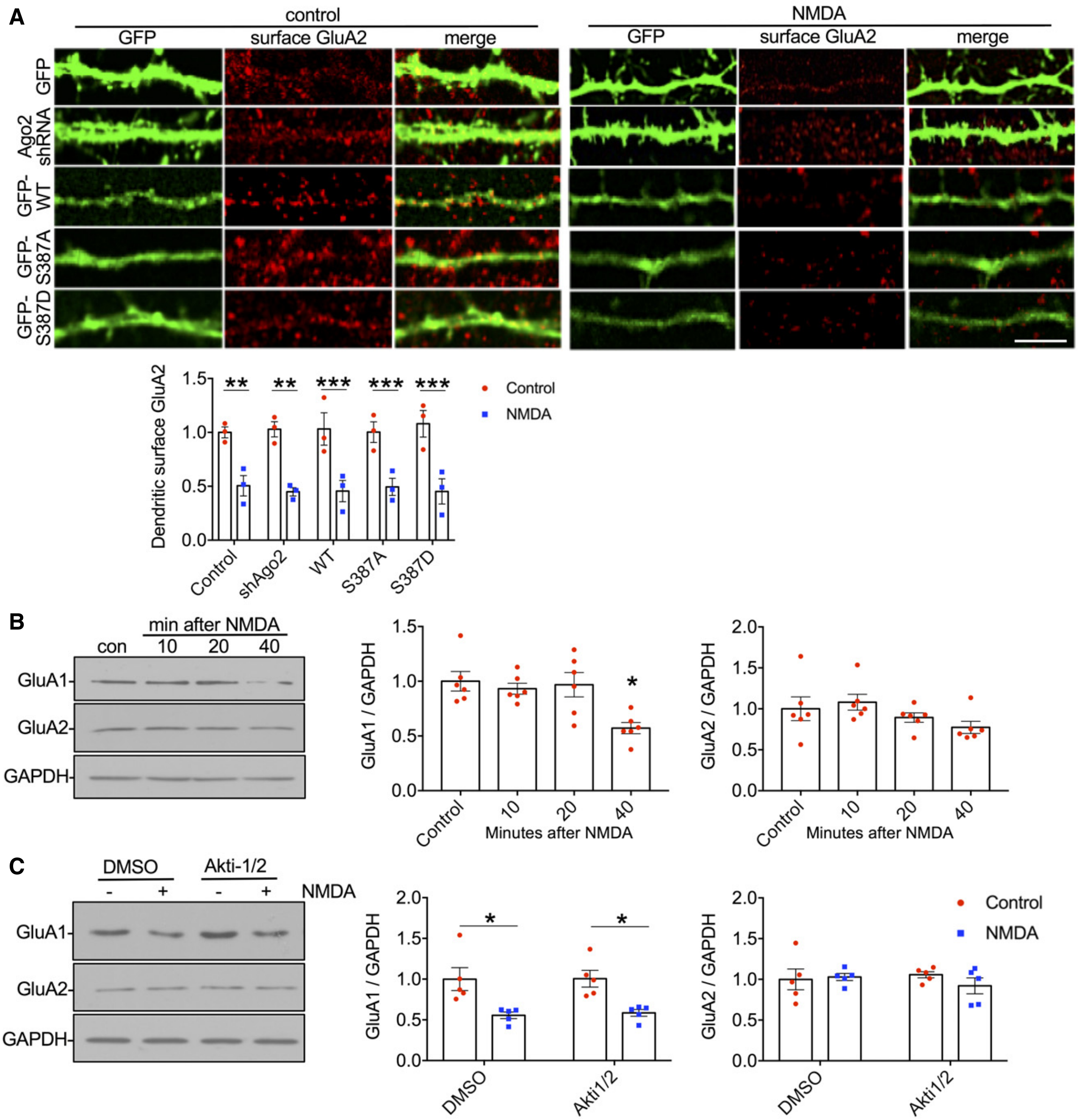


**Figure EV5. Ago2 phosphorylation at S387 is not required for NMDA-induced AMPAR internalisation (related to Fig 8).**

A NMDA-induced AMPAR internalisation is insensitive to Ago2 S387 mutations. Cortical neurons were transfected with molecular replacement constructs expressing Ago2 shRNA plus shRNA-resistant GFP-Ago2 (WT, S387A or S387D). Twenty minutes after NMDA or vehicle application, live cells were stained with GluA2 antibodies (red channel). GFP signal was maximised at acquisition so that dendrites could be effectively visualised. Graph shows surface GluA2 staining intensity in dendrites normalised to vehicle control. Scale bar = 20 μm; $n$ = 15 cells from three independent experiments. **$P < 0.01$; ***$P < 0.001$, two-way ANOVA, Bonferroni *post hoc* test. Error bars are SEM.

B Endogenous GluA1, but not GluA2, subunit levels are reduced in response to NMDAR stimulation. Cortical neuronal cultures were exposed to NMDA or vehicle for 3 min, and lysates were prepared 10, 20 or 40 min after NMDA washout and analysed by Western blotting. Graphs show quantification of GluA1 and GluA2 expression normalised to vehicle control; $n$ = 6. *$P < 0.05$; one-way ANOVA, Bonferroni *post hoc* test. Error bars are SEM.

C NMDAR-dependent decrease in GluA1 is Akt-independent. Cortical neuronal cultures were treated with Akti-1/2 20 min before NMDA or vehicle application, and lysates were prepared 40 min after NMDA washout and analysed by Western blotting. Graph shows quantification of GluA1 expression normalised to vehicle control; $n$ = 5. *$P < 0.05$; one-way ANOVA, Bonferroni *post hoc* test. Error bars are SEM.

Source data are available online for this figure.
