# Supplementary figures and images for: NMDAR‐dependent Argonaute 2 phosphorylation regulates miRNA activity and dendritic spine plasticity
Source: EMBO J. 2018 Apr 30;37(11):e97943. doi: 10.15252/embj.201797943 (PMC5983126; doi:10.15252/embj.201797943)

Figure EV1

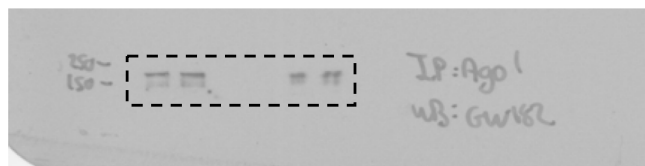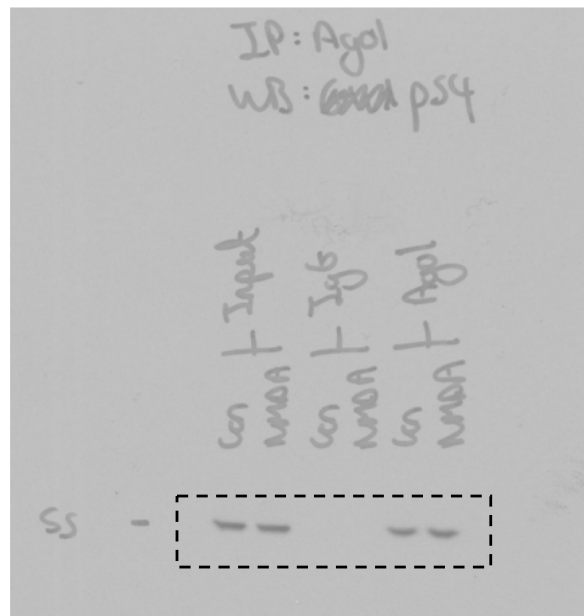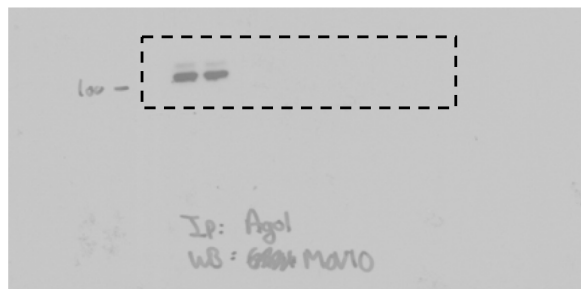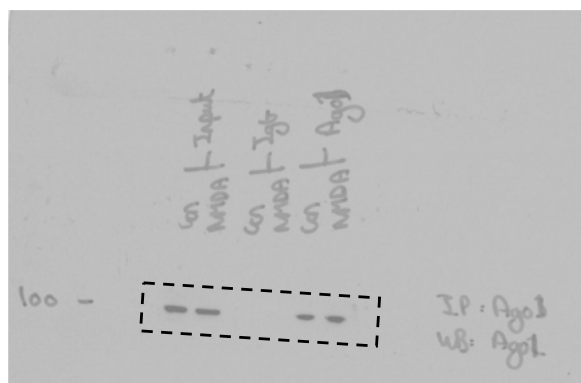

Supplement: Supplementary file 3 — Source Data for Expanded View and Appendix [file EMBJ-37-e97943-s008.zip › Figure_EV1.pdf]

Figure EV2

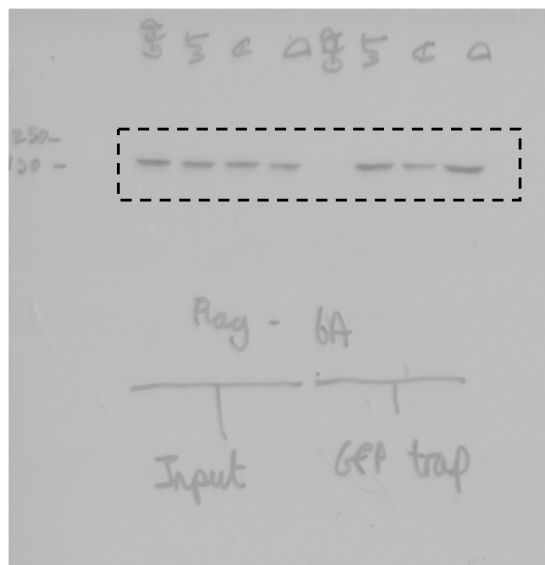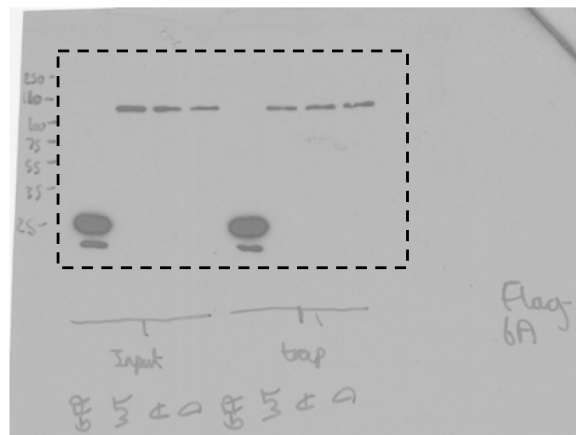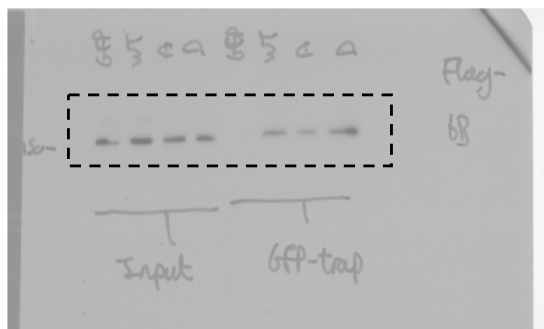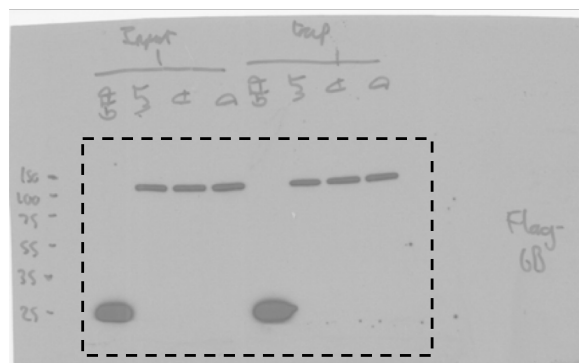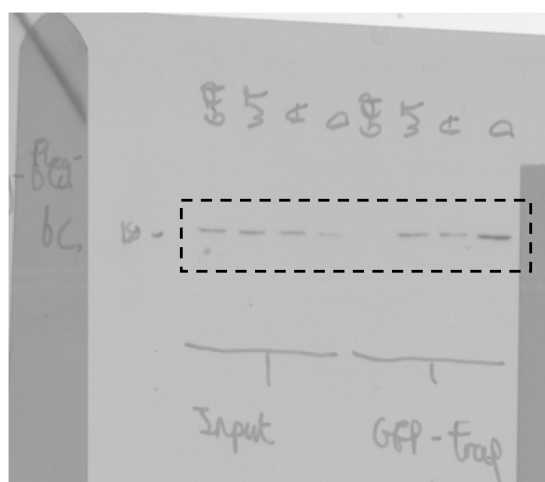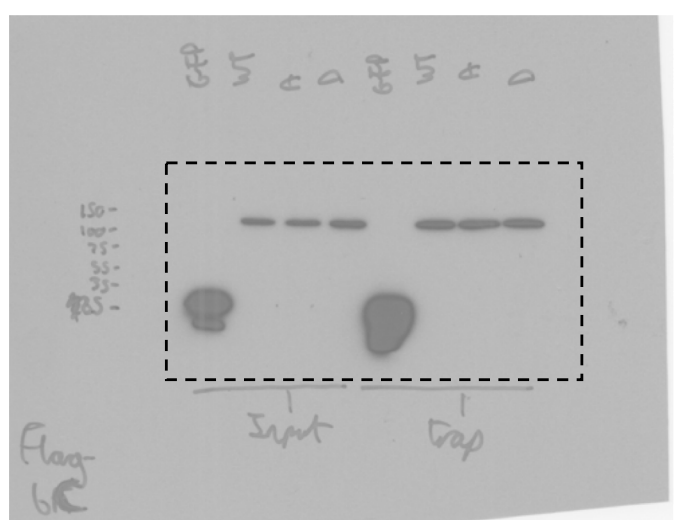

Supplement: Supplementary file 3 — Source Data for Expanded View and Appendix [file EMBJ-37-e97943-s008.zip › Figure_EV2.pdf]

Figure EV4

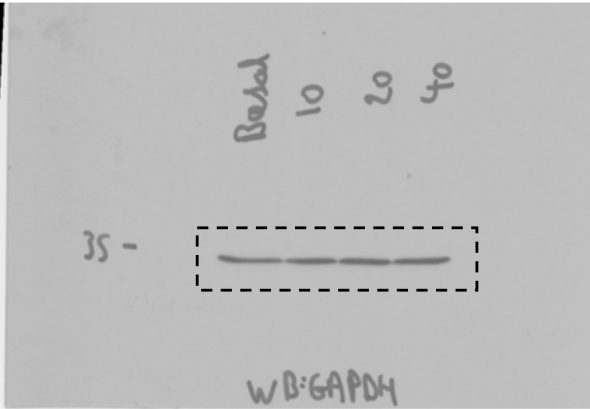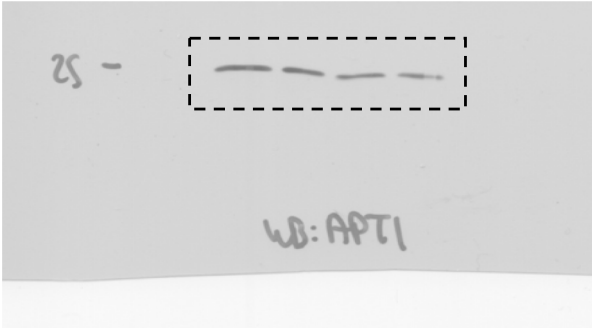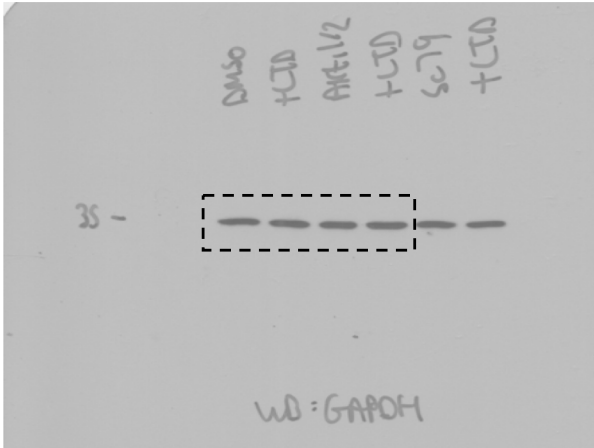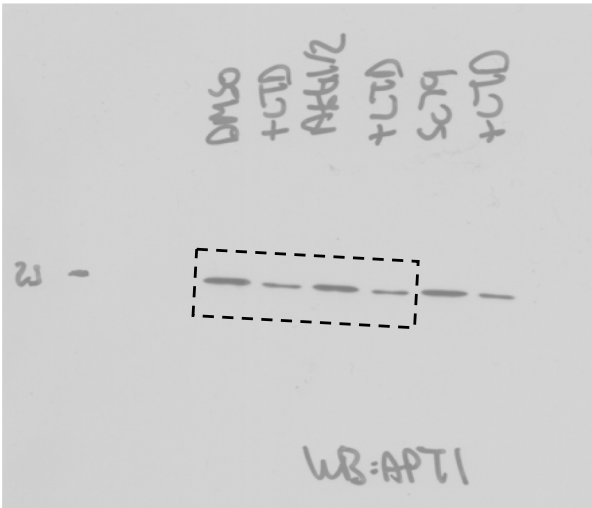

Supplement: Supplementary file 3 — Source Data for Expanded View and Appendix [file EMBJ-37-e97943-s008.zip › Figure_EV4.pdf]

Figure EV5

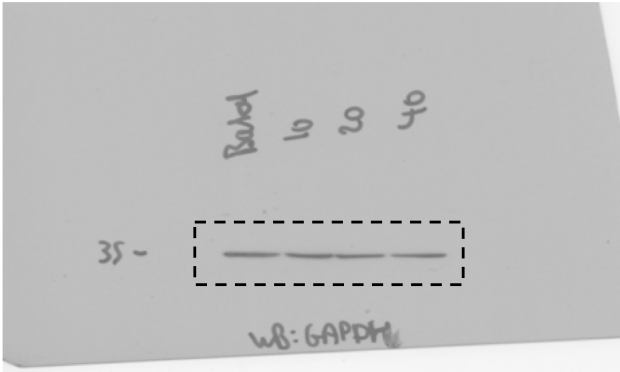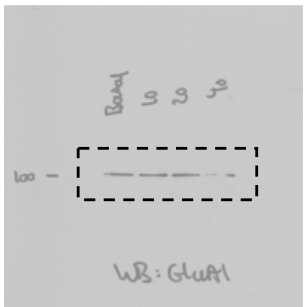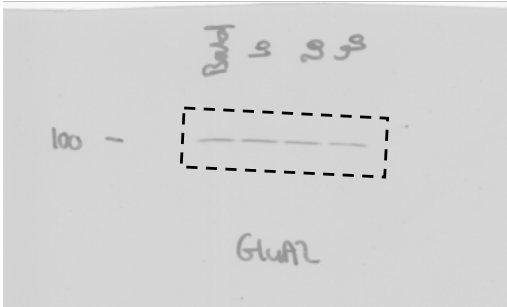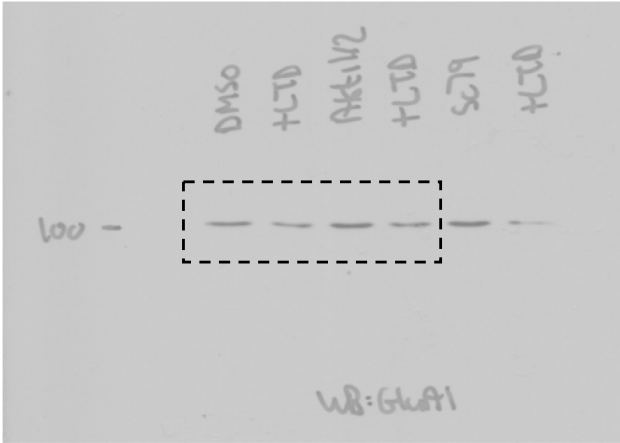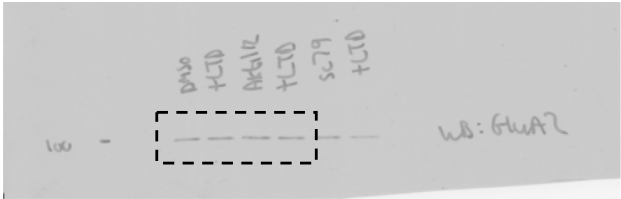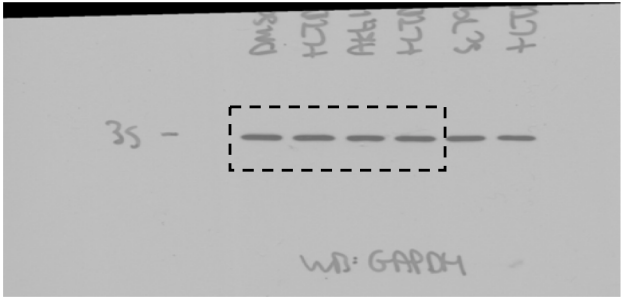

Supplement: Supplementary file 3 — Source Data for Expanded View and Appendix [file EMBJ-37-e97943-s008.zip › Figure_EV5.pdf]

## Appendix Fig S2

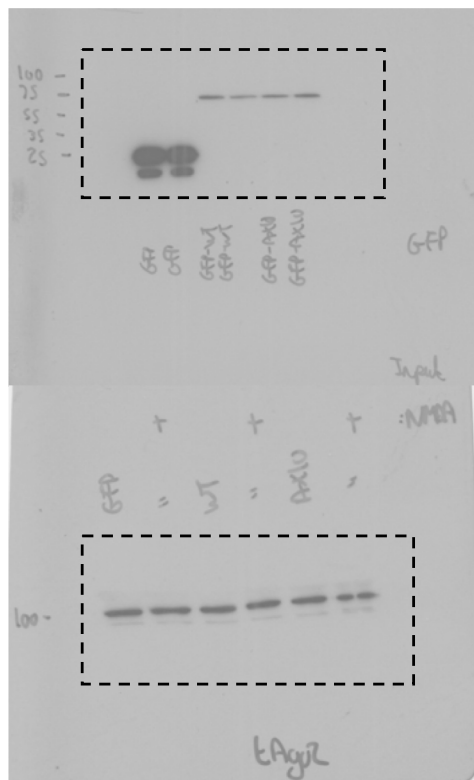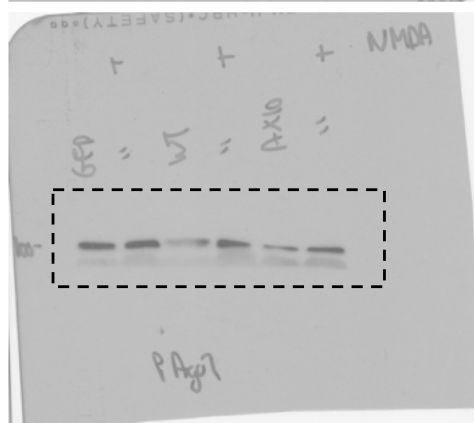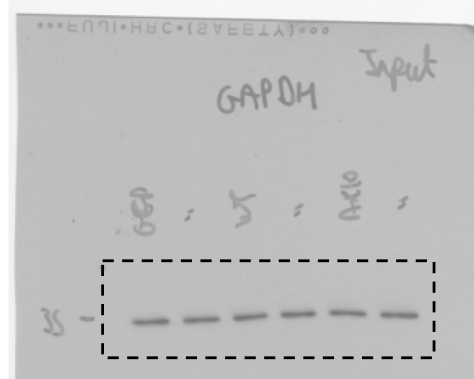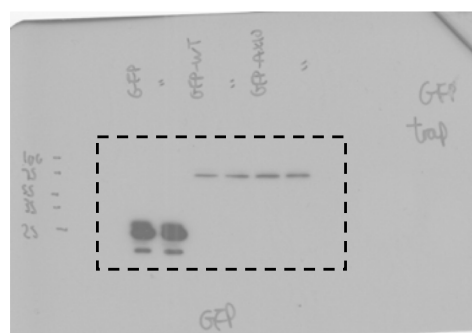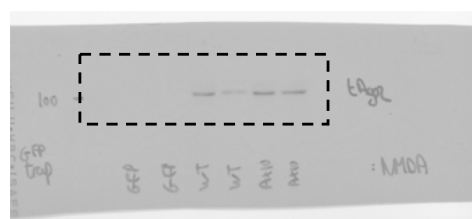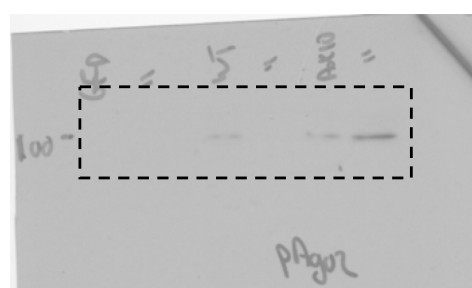

Supplement: Supplementary file 3 — Source Data for Expanded View and Appendix [file EMBJ-37-e97943-s008.zip › Appendix_Fig_S2.pdf]

# Appendix Fig S3

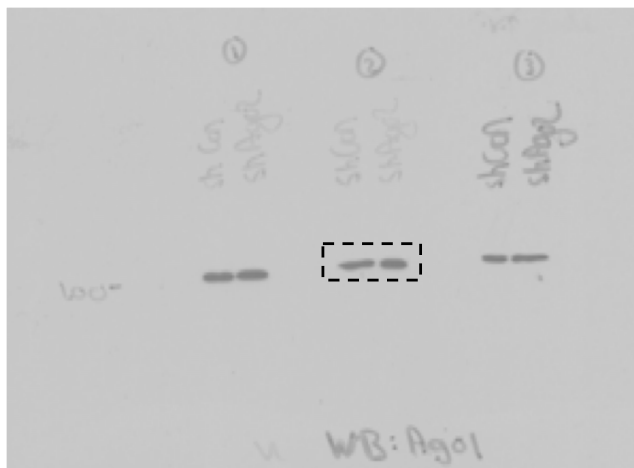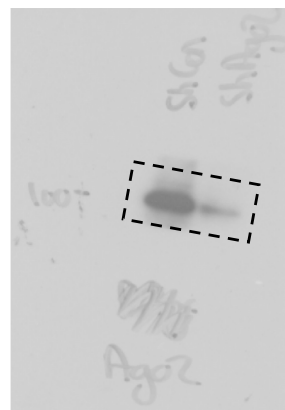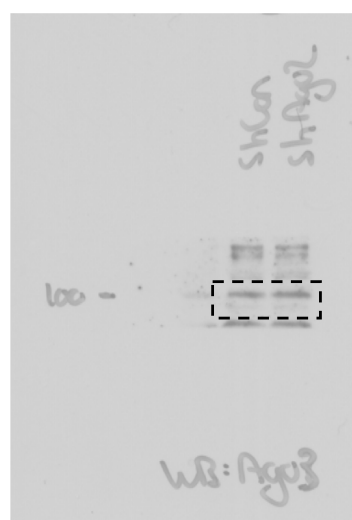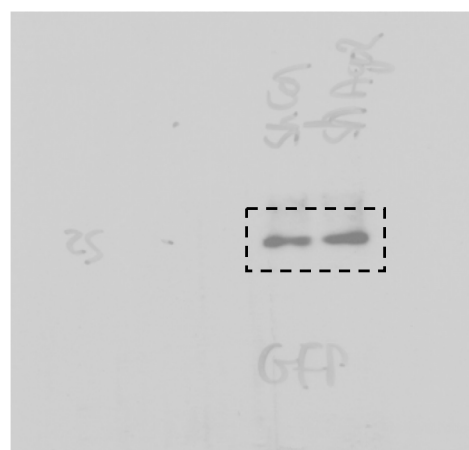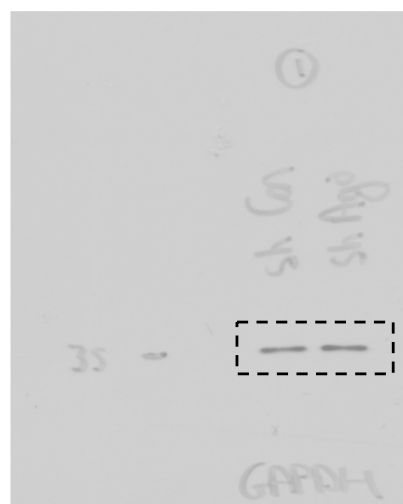

Supplement: Supplementary file 3 — Source Data for Expanded View and Appendix [file EMBJ-37-e97943-s008.zip › Appendix_Fig_S3.pdf]

Figure 1A

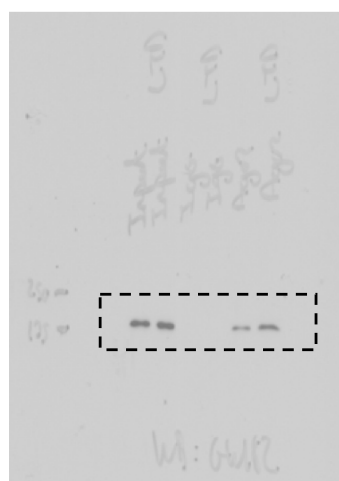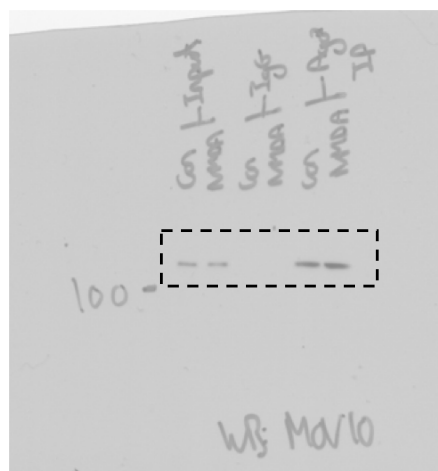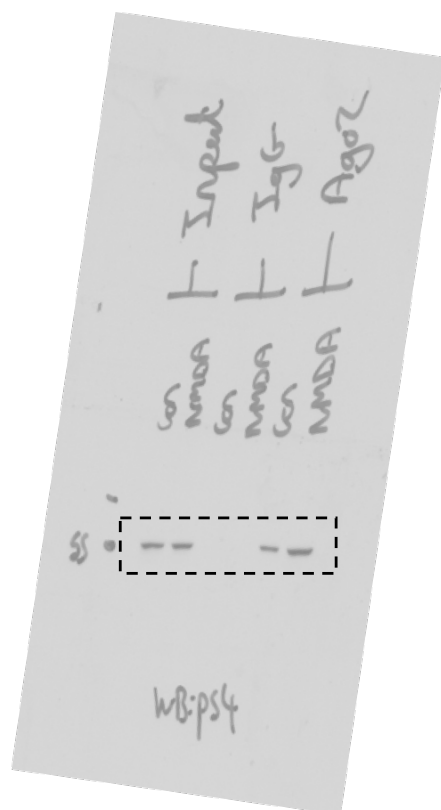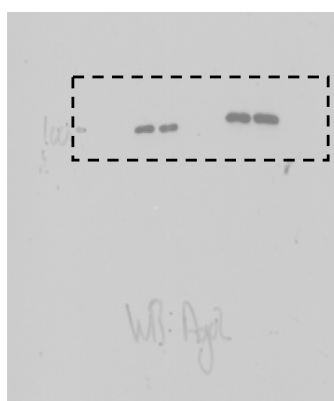

Supplement: Supplementary file 5 — Source Data for Figure 1 [file EMBJ-37-e97943-s003.pdf]

Figure 2A

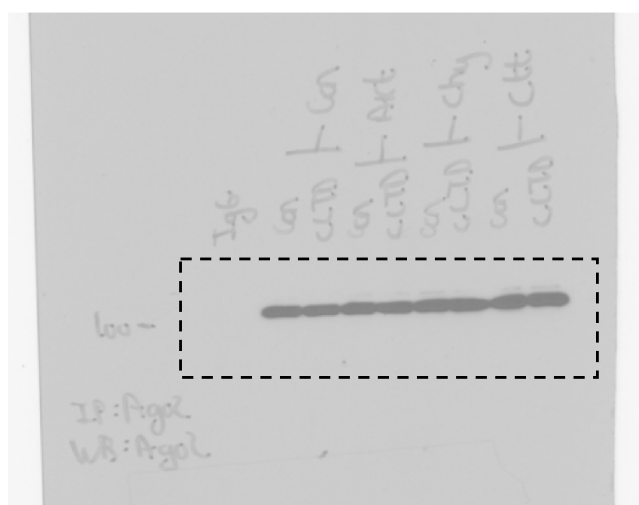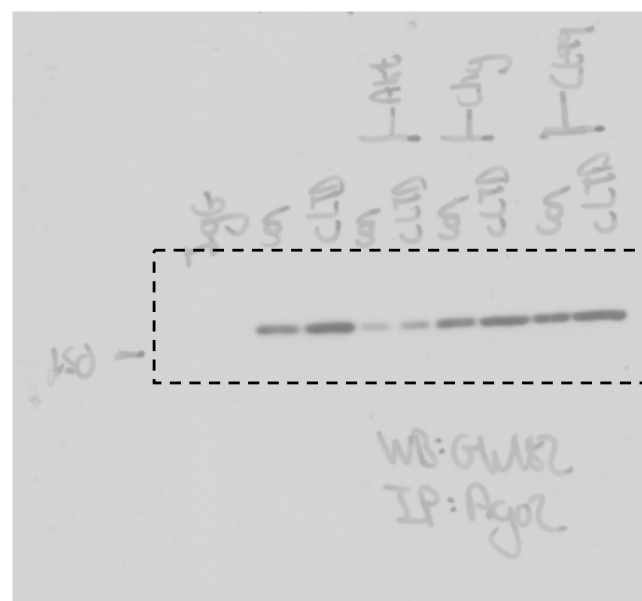

Figure 2B

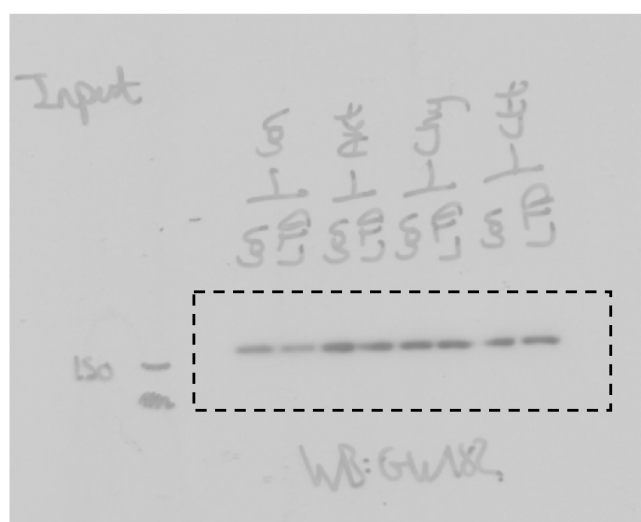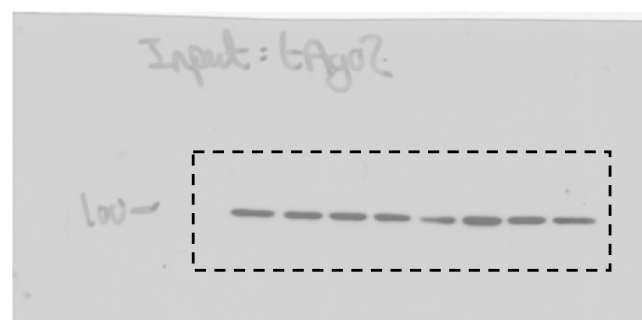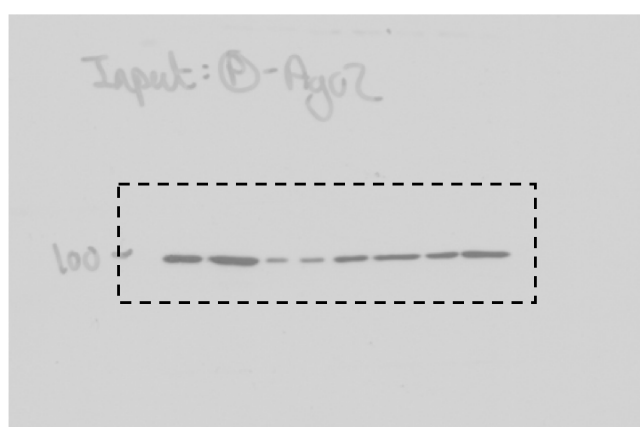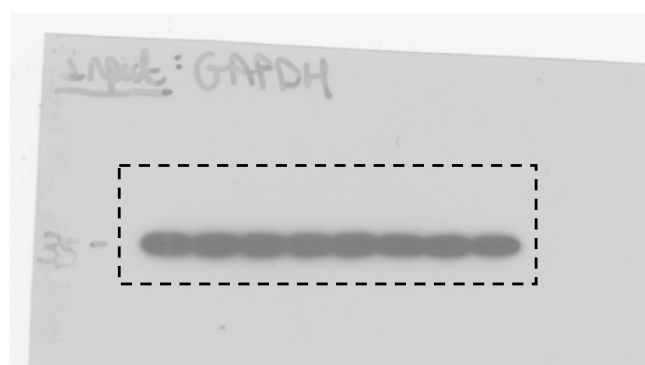

Figure 2C

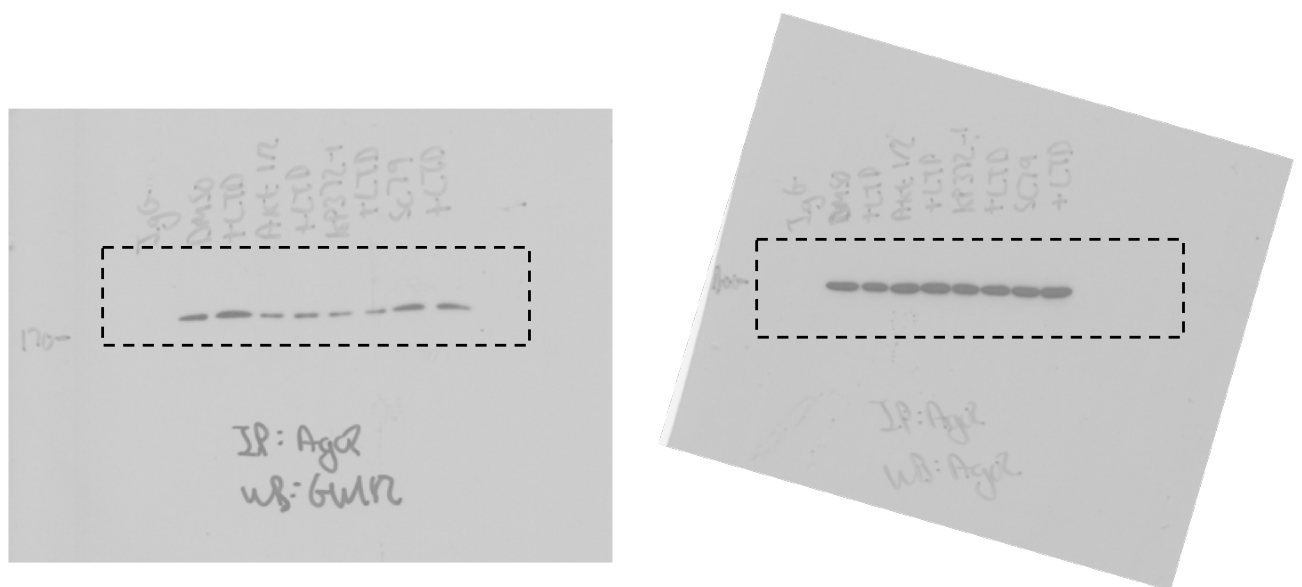

Figure 2D

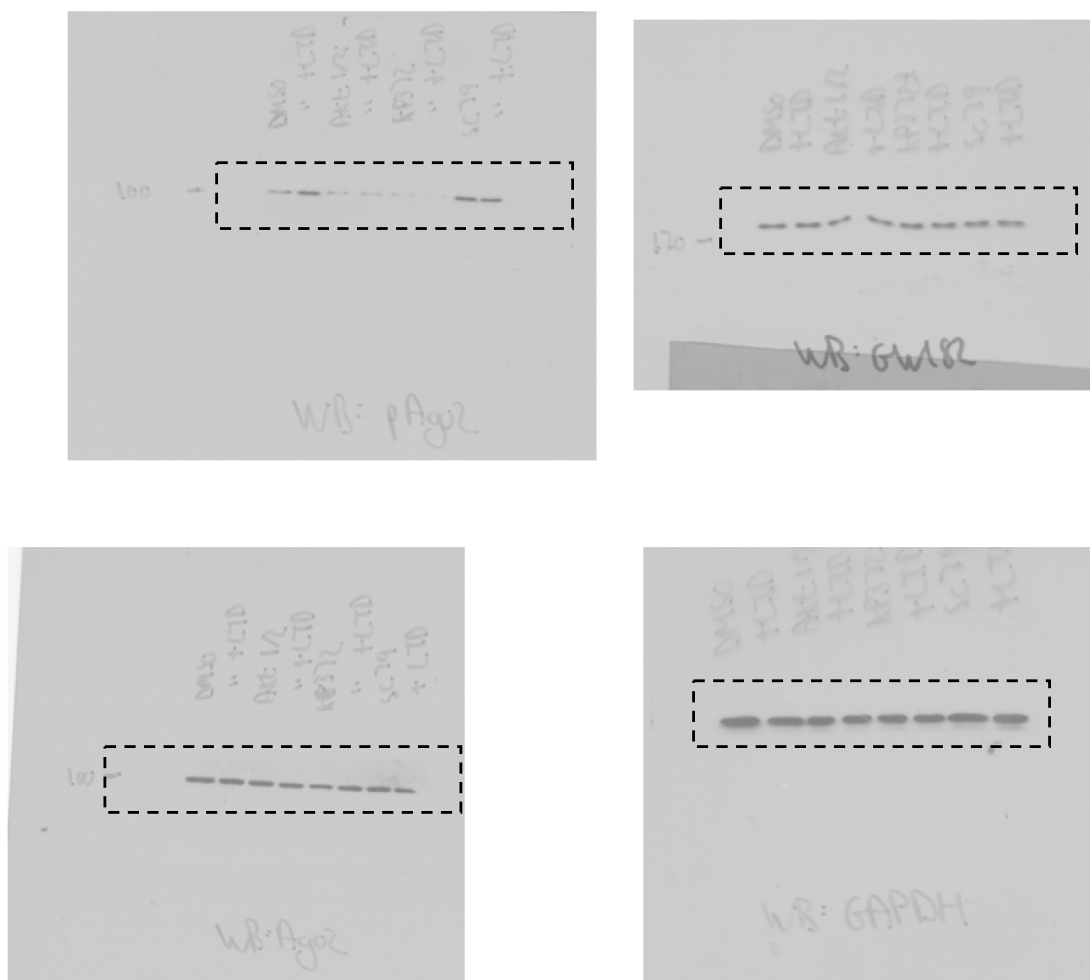

Figure 2E

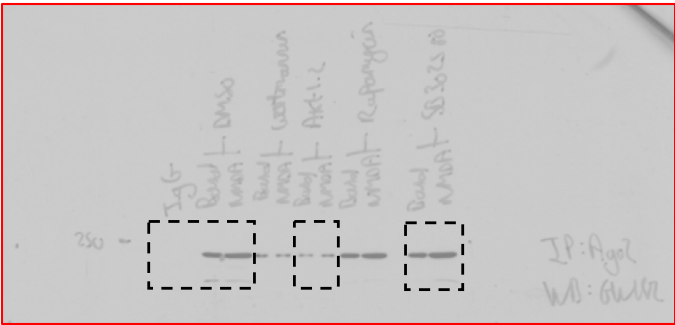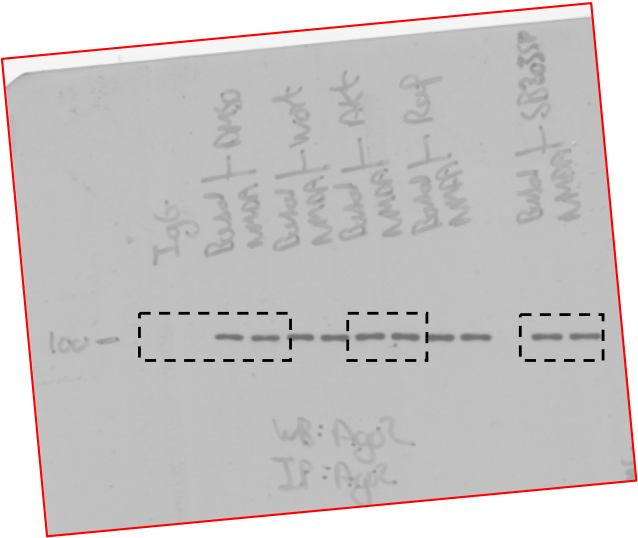

Figure 2F

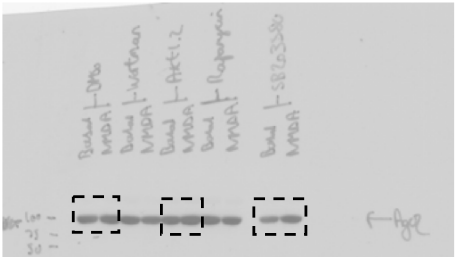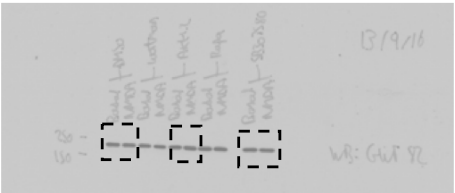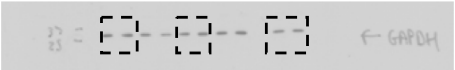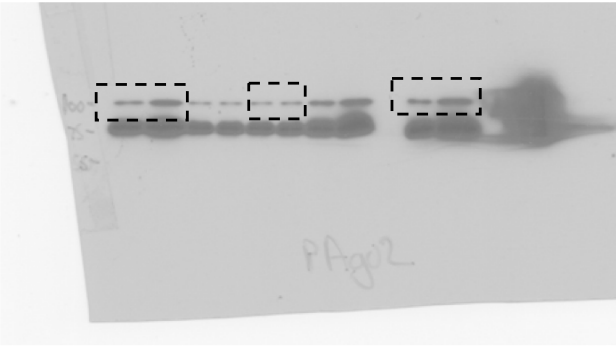

Supplement: Supplementary file 6 — Source Data for Figure 2 [file EMBJ-37-e97943-s004.pdf]

Figure 3A

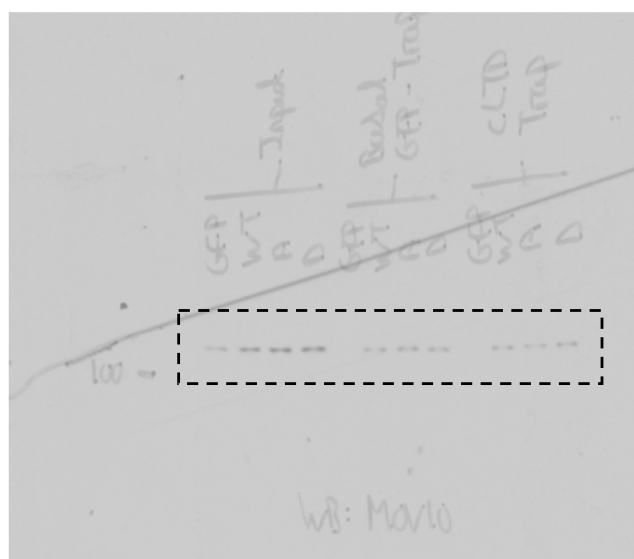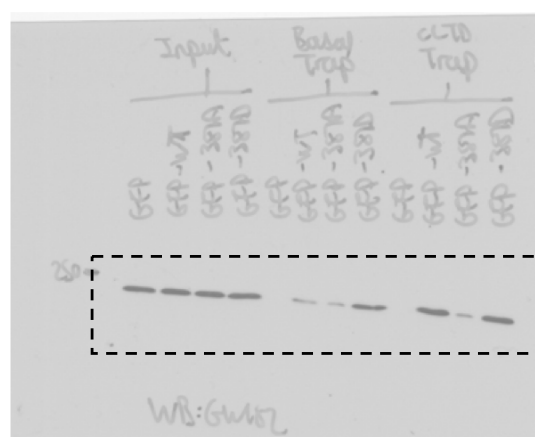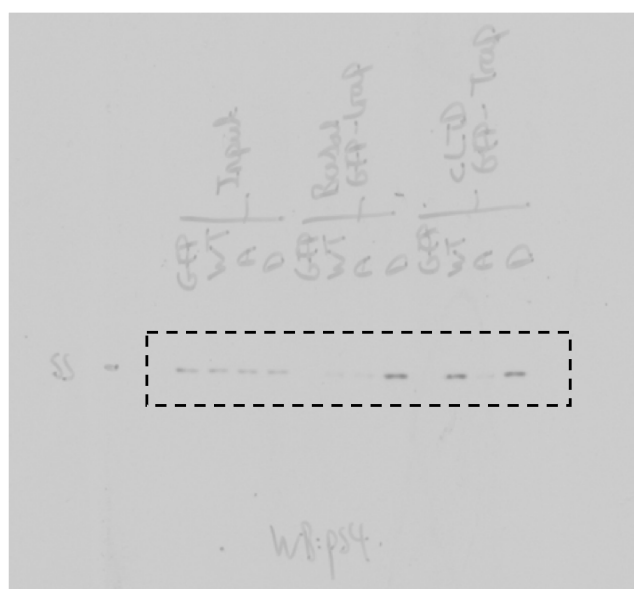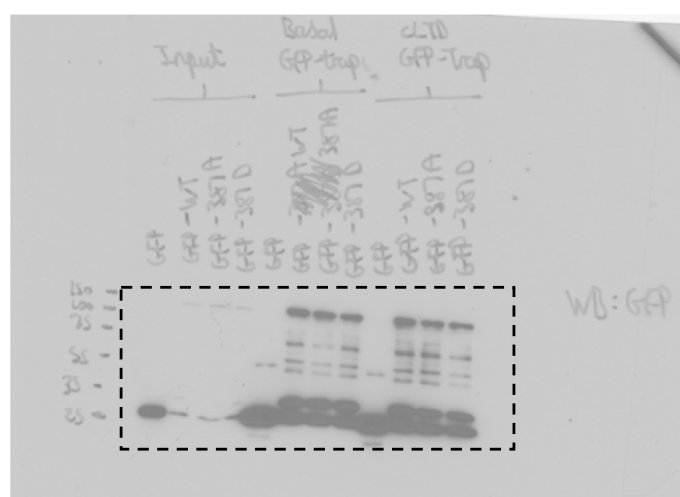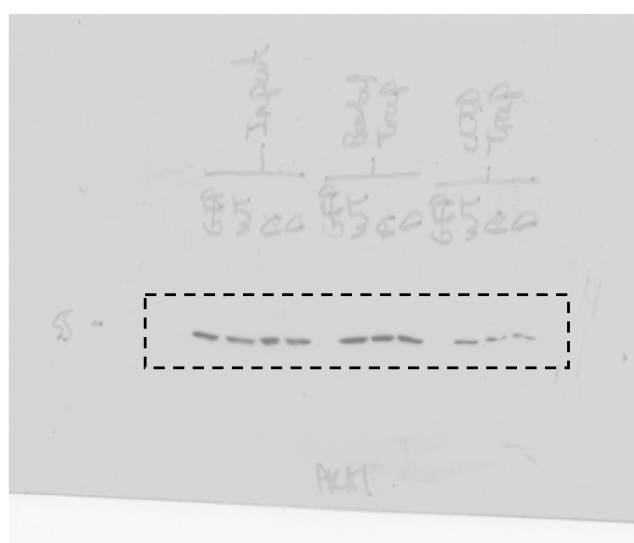

Supplement: Supplementary file 7 — Source Data for Figure 3 [file EMBJ-37-e97943-s005.pdf]

Figure 4A

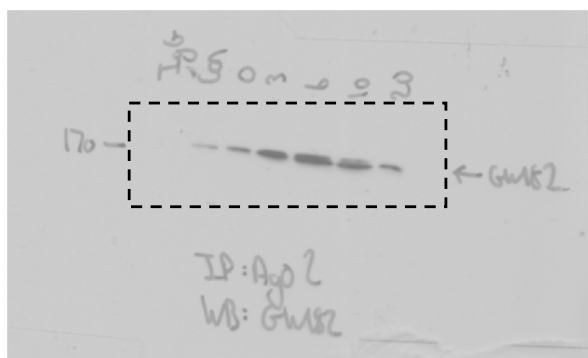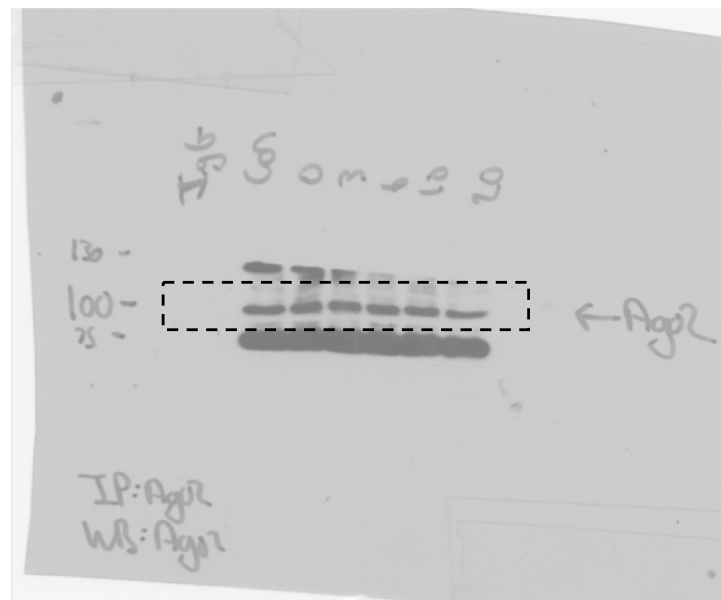

Figure 4B

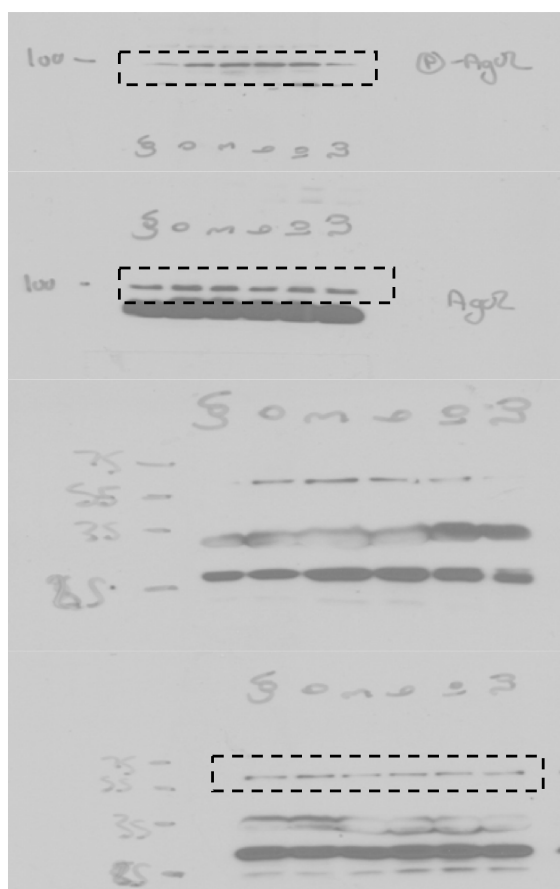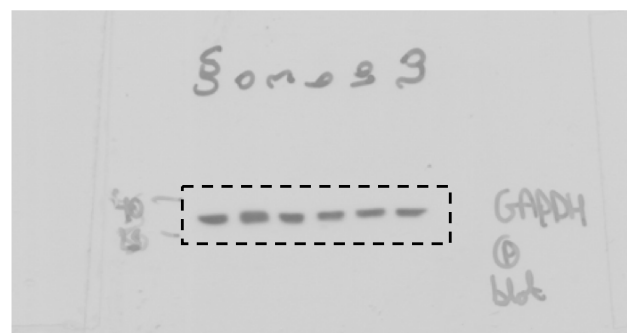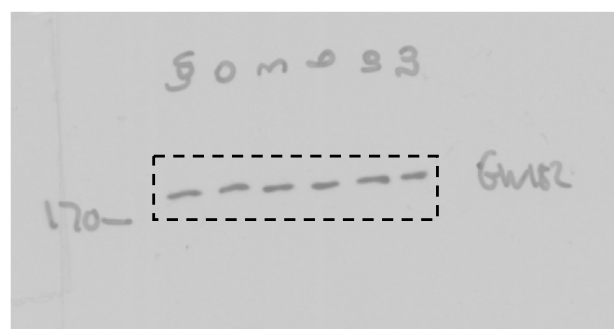

Supplement: Supplementary file 8 — Source Data for Figure 4 [file EMBJ-37-e97943-s006.pdf]

Figure 7A

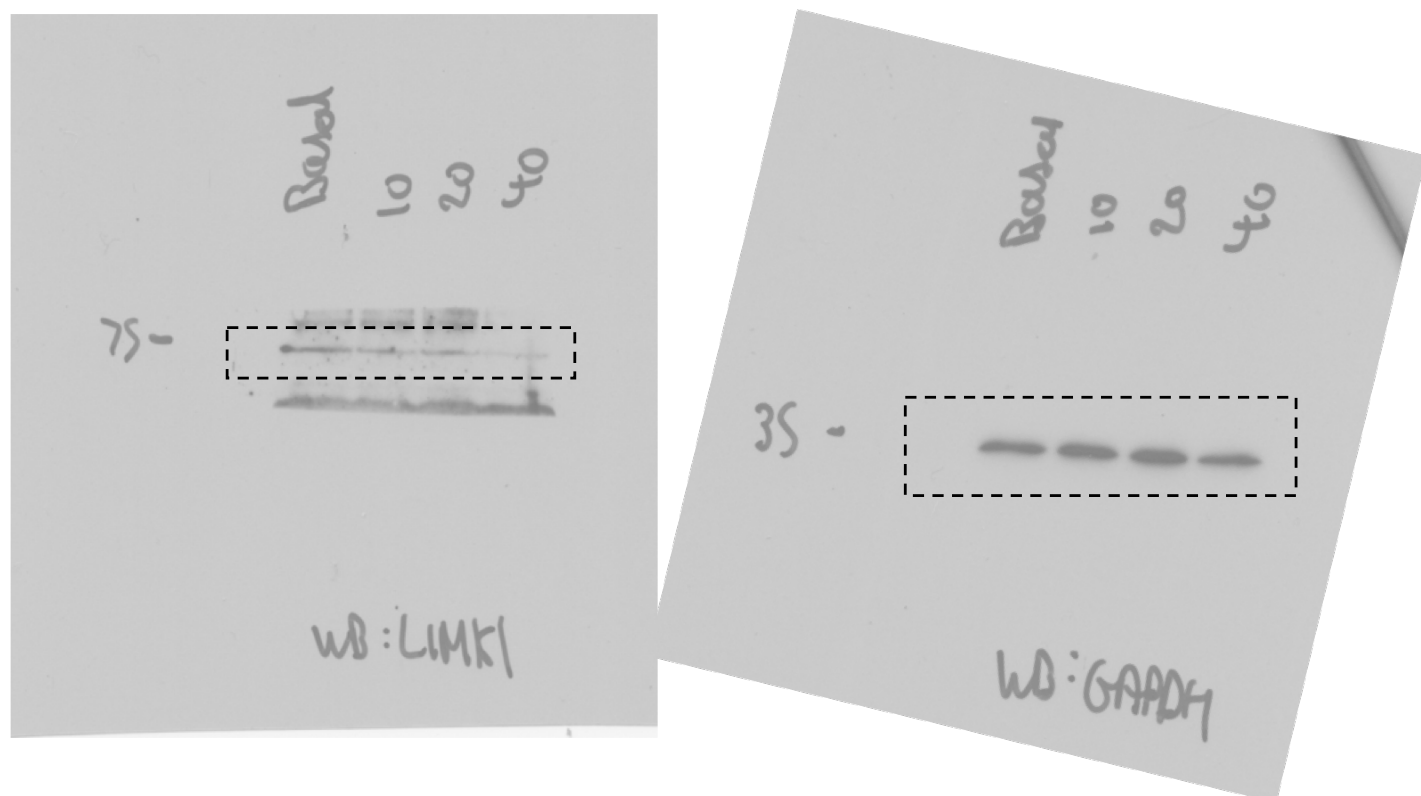

Figure 7B

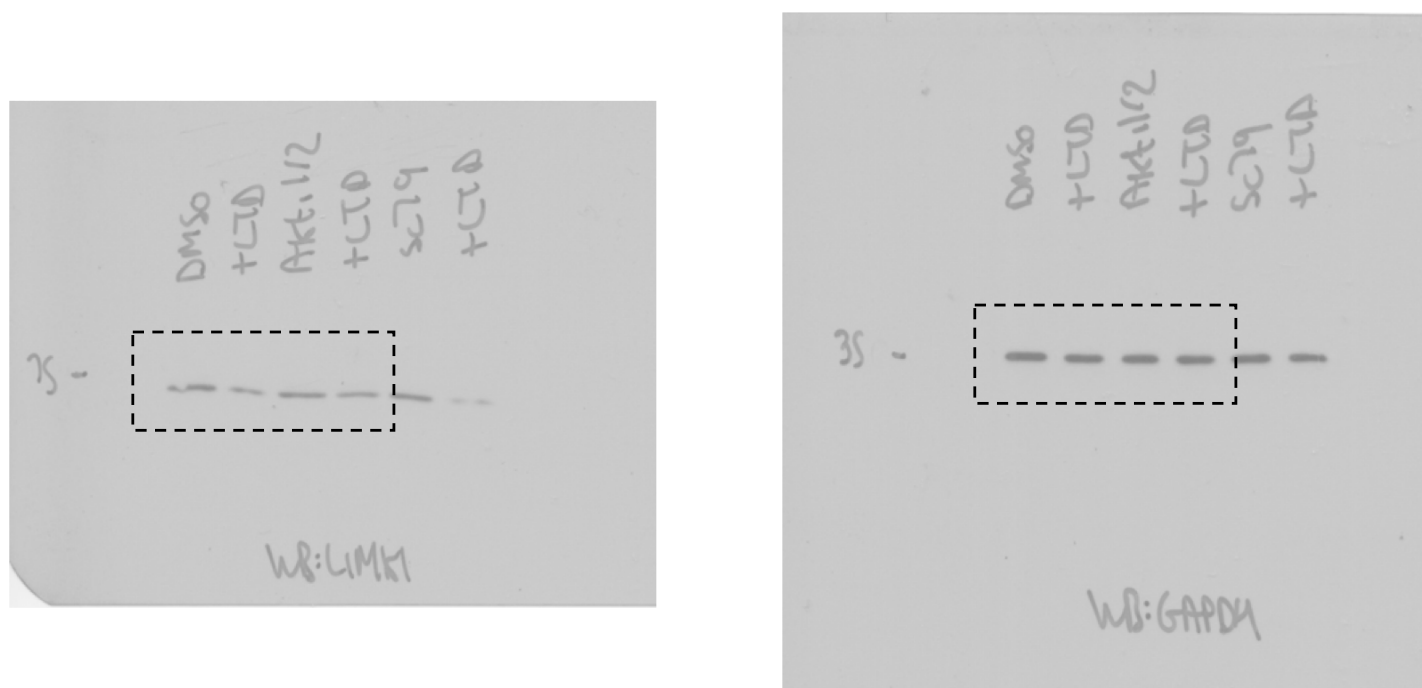

Supplement: Supplementary file 9 — Source Data for Figure 7 [file EMBJ-37-e97943-s007.pdf]
